# A description of sodium valproate, lamotrigine and levetiracetam consumption in the Western Cape public sector

**DOI:** 10.4102/safp.v64i1.5402

**Published:** 2022-06-02

**Authors:** Nicole Keuler, Yasmina Johnson, Renier Coetzee

**Affiliations:** 1 School of Pharmacy, Faculty of Natural Science, University of the Western Cape, Bellville, South Africa; 2 Department of Health, Western Cape Government, Cape Town, South Africa; 3 Provincial Pharmacy and Therapeutics Committee, Western Cape Government, Cape Town, South Africa

**Keywords:** sodium valproate, levetiracetam, lamotrigine, consumption, epilepsy

## Abstract

**Background:**

The rational use of medicine is fundamental to ensure effective and safe patient medicine treatment, and hence, should be monitored. Undisputable evidence exists for the teratogenic risk factors associated with sodium valproate. Consequently, the Western Cape Department of Health introduced a policy (2019) recommending alternatives for valproate in women of childbearing age, including lamotrigine or levetiracetam as alternatives for patients on antiretrovirals. This study aimed to describe the change in the consumption of valproate, lamotrigine and levetiracetam after a policy implementation in public sector health facilities of the Western Cape, South Africa.

**Methods:**

This observational study followed a quasi-experimental design. Consumption data from the Cape Medical Depot over the period 01 April 2018 to 31 March 2020 were analysed retrospectively. Consumption was presented as a defined daily dose (DDD) per 1000 population per quarter for sodium valproate, levetiracetam and lamotrigine for the Western Cape province, urban and rural areas. Consumption 12 months before was compared with consumption 12 months after policy implementation.

**Results:**

Post-policy implementation, valproate consumption remained unchanged provincially (3.3%; *p* = 0.255), in urban (7.8%; *p* = 0.255) and rural (1.5%; *p* = 0.701) areas. Lamotrigine consumption increased significantly provincially (30.7%; *p* = 0.020) and in urban areas (54.5%; *p* = 0.002); however, rural (26.1%; *p* = 0.108) areas did not show significant change. Provincially, valproate consumption remained substantially higher (209 DDDs/1000 population per quarter) compared with lamotrigine consumption (32.22 DDDs/1000 population per quarter).

**Conclusion:**

In the Western Cape public sector, the consumption of sodium valproate remained unchanged 12 months after policy implementation. Although there were significant increases in lamotrigine and levetiracetam consumption, the consumption was considerably less compared with sodium valproate consumption.

## Background

According to the World Health Organization (WHO), national medicine policies should form an integral part of the healthcare system to warrant safe and effective medicine use.^1^ A health policy is defined as a law or regulation implemented to promote public health by influencing systems, organisation change and individual behaviour. It is an essential function in health care.^2^ In addition, these policies should be continuously revised to ensure the rational use of medicine. As per the WHO, the rational use of medicines requires that patients receive medications appropriate to their clinical needs, in dosages that are personalised, for an appropriate time at a cost that is affordable to both the community and the patient.^3^ Approximately 50% of medicines globally are prescribed, dispensed or sold inappropriately. Inappropriate use of medicines may result in health risks.^4^

In the Western Cape Government Health (WCGH), medicine prescribing is guided by the South African Essential Medicine List (EML) and the Standard Treatment Guidelines (STGs) for Hospital level^5^ and Primary Healthcare level^6^, as well as the Western Cape Provincial Code List (PCL) – that is a list of medicines with relevant prescribing levels. The Provincial Pharmacy and Therapeutics Committee maintains the PCL, aims to align national (STG & EML) and provincial policy as far as possible, and provides additional medicine policy guidance to the province when required.

Sodium valproate is a first-generation antiepileptic, highly protein bound with a narrow therapeutic index. Valproate is metabolised by cytochrome (CYP) P450 enzymes in the liver but can simultaneously inhibit CYP450 enzymes and uridine 5′-diphosphate-glucuronosyltransferase (UGT), which can lead to an increase in the concentration of lamotrigine and phenobarbital because of a reduced clearance.^7^ Valproate is primarily used in the treatment of epilepsy but can also be used in the treatment of bipolar disorder and migraine. Other first-generation anti-epileptics include phenytoin, phenobarbital and carbamazepine, and second-generation anti-epileptics include lamotrigine, levetiracetam, topiramate and gabapentin. Lamotrigine is metabolised by UGT1A4, which can be induced by ritonavir and lead to drug–drug interactions requiring a dose increase of lamotrigine with co-administration of ritonavir containing anti-retroviral (ARV) therapy.^8,9^ However, sodium valproate has less drug–drug interactions with ARVs. Sodium valproate has the highest teratogenic risk compared with levetiracetam and lamotrigine.^10,11^ The mechanism of action of teratogenicity is thought to be multifactorial, including reduced folic levels, oxidative stress causing apoptosis and interfering with DNA synthesis.^12^ The risk to the fetus does not only include major congenital malformations but also neurodevelopmental problems.^10,11^ Various media releases^13,14,15^ warned about the teratogenic harms of sodium valproate after multiple studies published evidence of this risk.^10,11,16,17,18,19^

The WCGH (public sector) provides health care to more than three-quarters of the population (78%) who resides in the Western Cape province.^20^ In the WCGH, over a 3-year period, sodium valproate remained the most frequently prescribed antiepileptic in women of childbearing age (WOCBA) compared with other anti-epileptics and mood stabilisers available in the public sector (carbamazepine, risperidone, phenytoin, lamotrigine, olanzapine, lithium and levetiracetam), that is, 43.2% in 2015, 45.4% in 2016 and 44.8% in 2017.^21^ Furthermore, sodium valproate was the most frequently prescribed antiepileptic in WOCBA primarily for the treatment of epilepsy in the retroviral disease positive population.^21^ As a consequence of safety warnings and the high use of sodium valproate, the WCGH introduced a policy on the use of valproate in epilepsy in May 2019, which suggested alternatives and promoted the safe use of valproate in epilepsy in WOCBA.^22^ The policy was shared electronically with healthcare professionals in the public sector and accompanied by a series of information or training sessions for relevant staff. Because of concerns of serious lamotrigine side effects (e.g. Steven-Johnson-like rash), a tool to monitor the safe initiation of lamotrigine was implemented concurrently.

The policy outlined the reason for switching to alternative therapy, provided guidance in instances where sodium valproate remained the preferred choice, and provided guidance on how to taper off sodium valproate and initiate an alternative therapy. The policy also outlined details on cases to be referred, as well as specialists (e.g. family physicians) involvement in the safe initiation of lamotrigine. Counselling and risk acknowledgement forms for health professionals and patients formed part of the policy. The epilepsy policy recommended against the use of sodium valproate in WOCBA. However, sodium valproate may be prescribed in male and female patients incapable of becoming pregnant.^22^ Sodium valproate and lamotrigine are preferred anti-epileptics in patients on ARVs because of minimal interactions with ARVs.^5^ For these patients, sodium valproate was preferred in the WCGH as it did not require titration on initiation as does lamotrigine. The new epilepsy policy recommended lamotrigine as the first-line alternative to sodium valproate for patients on ARVs and levetiracetam where lamotrigine would not be suitable. Alternative therapy options for sodium valproate included lamotrigine, levetiracetam, carbamazepine, phenytoin and phenobarbitone as clinically appropriate or referral of more complex cases.^22^ It was expected, therefore, that the policy should affect a decrease in the consumption of valproate and an increase in the consumption of lamotrigine and levetiracetam.

The aim of this research study was to report on the change in the consumption of sodium valproate, lamotrigine and levetiracetam in WCGH facilities after the implementation of the policy on the safe use of valproate in epilepsy in 2019.^22^

## Method

### Study design

This was an observational study following a quasi-experimental study design. We compared the consumption data of sodium valproate, lamotrigine and levetiracetam by WCGH facilities, pre- and post-policy implementation (intervention).

### Population and data analysis

The Cape Medical Depot (CMD) is a wholesaler that supplies pharmaceuticals to all public sector healthcare facilities in the province, except the three tertiary hospitals. For the study, quantities of sodium valproate, levetiracetam and lamotrigine issued to facilities from the CMD, over the period 01 April 2018 to 20 March 2020, were retrieved using the CMD electronic Medical Supply Administration System (MEDSAS). MEDSAS provides medicine consumption information per facility, district or substructure and province.

**FIGURE 1:**
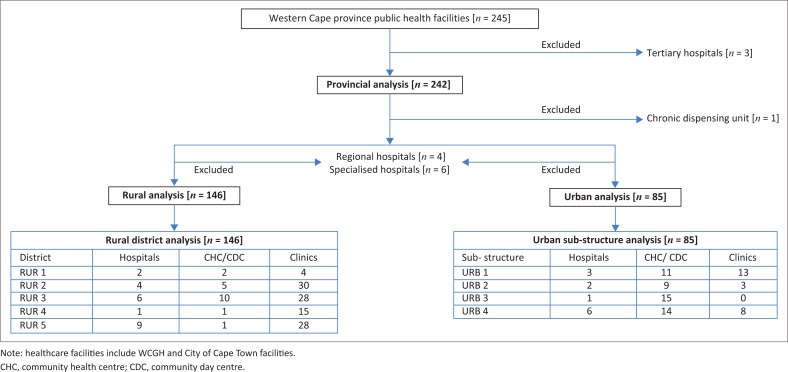
Illustration of the public healthcare facilities included in the consumption analyses of valproate, lamotrigine and levetiracetam.

The Western Cape Government Health reshaped public health services to focus on primary-level services, with the aim of 89.0% of acute contacts at level 1, 8.0% at level 2 and 3.0% at level 3.^23^ Furthermore, 99.5% of chronic contacts are to be serviced at level 1 and 0.5% at level 2. Level 3 (tertiary) is reserved for when sub-specialist consultation is required with a repeat of medicines referred to primary care services.^23^ Thus, the data analysis for the provincial level included all Western Cape public sector healthcare facilities (primary health care [PHC] facilities, district-, regional-, specialist hospitals and the chronic dispensing unit [CDU]), but excluded the tertiary hospitals (i.e. < 1% of patient contacts) as the tertiary hospitals’ consumption do not appear on the CMD MEDSAS. In order to investigate regional or geographical-specific variations, consumption within urban, rural, districts and substructures was analysed. For this sub-analysis, all healthcare facilities in these respective areas were included; however, the CDU, regional and specialised hospitals were excluded as these institutions provide services across geographical platforms, and thus, are not specific for geographical analysis. Figure 1 shows the health facilities included and excluded in the various consumption analyses performed on sodium valproate, lamotrigine and levetiracetam.

Consumption was presented as a defined daily dose (DDD)/1000 population per quarter. The DDD of a medicine is an international measurement defined as the assumed average daily maintenance dose for a medicine used for its main indication in adults. Analysing data as per DDDs makes it possible to combine different strengths, pack sizes and formulations of a medicine into a single value of consumption in order to assess changes in medication utilisation over time, determine the impact of an intervention or compare consumption across geographical areas and countries. The WHO-recommended DDDs for sodium valproate (DDD: 1.5 g), lamotrigine (DDD: 0.3 g) and levetiracetam (DDD: 1.5 g) were utilised.^24^ Per antiepileptic, the strengths, pack sizes and quantities issued per quarter per facility were extracted from the CMD MEDSAS report. The population circular^25^ was used to calculate the population for the province, urban, rural, district and substructure areas for the period of analysis. The data were analysed per financial quarter for four quarters before policy implementation (Q1: 01 April 2018 – 30 June 2018 up to Q4: 01 January 2019 – 31 March 2019) and for four quarters after policy implementation (Q5: 01 April 2019 – 30 June 2019 up to Q8: 01 January 2020–31 March 2020).

Consumption was calculated as follows:

$$\begin{array}{l} \text{DDDs}/1000\text{population}/\text{quarter}=\\ {}[\text{antiepileptic strength}\times \text{packsize}\times \\ \text{quantity of packs issued per quarter}/\\ \text{DDD of antiepileptic}\times \text{population}]\times 1000 \end{array}$$ (Eqn 1)

The paired *t*-test was used to compare the mean quarterly consumption before and after the intervention. A confidence interval (CI) of 95% was reported with statistical significance set as *p* < 0.05.

### Ethical considerations

The study did not include any personal health information, and therefore, no consent was required. The dataset was received from the Cape Medical Depot and approval for analysis, and reporting was approved by the Chief Directorate Emergency and Clinical services, as well as the Directorate Pharmacy Services for the Western Cape Government Health (No. 18/2/18/7).

## Results

The average quarterly consumption of sodium valproate, lamotrigine and levetiracetam in the Western Cape province, urban (also known as Cape Metropole) and rural areas over the 12 months prior and 12 months post the policy implementation, as well as the change in consumption post-policy implementation, are shown in Tables 1–3. In the Western Cape, consumption of sodium valproate (209 DDD/1000 population per quarter) remained substantially higher compared with lamotrigine (32 DDD/1000 population per quarter) and levetiracetam (1.3 DDD/1000 population per quarter).

**TABLE 1:** Mean consumption per quarter and change in consumption of sodium valproate pre- and post-policy implementation, 2018–2020.

Area	Pre-mean		Post-mean		Diff-mean		95% CI	*p*	Change (%)
	DDD/1000 pop	s.d.	DDD/1000 pop	s.d.	DDD/1000 pop	s.d.			
**Prov**	202.41	9.9	209.11	18.0	6.70	9.6	−8.50–21.90	0.255	3.3
**Urban**	136.88	5.5	147.51	16.5	10.63	15.1	−13.46–34.71	0.255	7.8
URB 1	158.05	12.7	165.42	28.0	7.38	24.3	−31.27–46.02	0.587	4.7
URB 2	149.71	16.8	160.29	14.1	10.58	25.7	−30.28–51.44	0.470	7.1
URB 3	132.43	7.2	142.61	14.2	10.18	11.0	−7.35–27.70	0.161	7.7
URB 4	110.7	4.8	124.89	16.0	14.19	13.3	−6.98–35.35	0.122	12.8
**Rural**	152.49	8.4	154.77	15.0	2.27	10.8	−14.87–19.41	0.701	1.5
RUR 1	202.52	15.0	170.35	20.3	−32.17	20.2	−64.33–0.15	0.050	−15.9
RUR 2	133.64	10.5	129.22	7.5	−4.43	8.2	−17.47–8.61	0.359	−3.3
RUR 3	219.03	33.8	224.66	44.6	5.63	28.8	−40.25–51.50	0.722	2.6
RUR 4	132.93	18.7	157.25	23.1	24.32	36.8	−34.20–82.84	0.278	18.3
RUR 5	105.60	13.3	109.23	22.5	3.64	14.0	−18.68–25.96	0.640	3.4

CI, confidence interval; DDD, defined daily dose; s.d., standard deviation; diff, difference; pop, population; Prov, province.

Pre-mean = Mean consumption per quarter pre-policy implementation; Post-mean = Mean consumption per quarter post-policy implementation. Diff-Mean = Mean difference between the post-policy consumption and the pre-policy consumption. Percentage (%) change = ([post-consumption – pre-consumption] ÷ pre-consumption) × 100.

**TABLE 2:** Mean consumption per quarter and change in the consumption of lamotrigine pre- and post-policy implementation, 2018–2020.

Area	Pre-mean		Post-mean		Diff-mean		95% CI	*p*	Change (%)
	DDD/1000 pop	s.d.	DDD/1000 pop	s.d.	DDD/1000 pop	s.d.			
**Prov**	24.66	2.3	32.22	5.5	7.57	3.3	2.31–12.83	0.020	30.7
**Urban**	14.91	2.6	23.03	3.4	8.12	1.6	5.64–10.60	0.002	54.5
URB 1	12.84	3.0	25.11	3.0	12.28	0.5	11.41–13.15	0.000	95.6
URB 2	12.64	2.6	21.37	1.9	8.73	3.3	3.40–14.05	0.014	69.1
URB 3	18.65	2.1	25.61	4.9	6.96	4.3	0.11–13.80	0.048	37.3
URB 4	15.11	3.7	19.97	6.0	4.86	4.1	−1.61–11.32	0.097	32.2
**Rural**	15.70	2.2	19.79	4.7	4.09	3.6	−1.65–9.83	0.108	26.1
RUR 1	10.46	4.3	13.27	5.5	2.81	8.4	−10.62–16.23	0.553	26.9
RUR 2	14.49	2.8	18.09	5.8	3.60	5.3	−4.80–11.99	0.266	24.8
RUR 3	19.13	2.1	23.47	2.5	4.33	1.1	2.57–6.09	0.004	22.7
RUR 4	12.20	1.3	18.76	5.3	6.56	6.3	−3.43–16.54	0.128	53.8
RUR 5	16.65	4.2	20.06	9.2	3.41	8.9	−10.75–17.57	0.499	20.5

CI, confidence interval; DDD, defined daily dose; s.d., standard deviation; pop, population; Diff, difference; Prov, provincial.

Pre-mean = mean consumption per quarter pre-policy implementation; Post-mean = mean consumption per quarter post-policy implementation. Diff-Mean = mean difference between the post-policy consumption and the pre-policy consumption. Percentage (%) change = ([post-consumption – pre-consumption] ÷ pre-consumption) × 100.

**TABLE 3:** Mean consumption per quarter and change in the consumption of levetiracetam pre- and post-policy implementation, 2018–2020.

Area	Pre-mean		Post-mean		Diff-mean		95% CI	*p*	Change (%)
	DDD/1000 pop	s.d.	DDD/1000 pop	s.d.	DDD/1000 pop	s.d.			
**Prov**	0.125	0.20	1.270	0.70	1.150	0.50	0.40–1.89	0.016	916.0
**Urban**	0.023	0.05	0.200	0.20	0.180	0.10	−0.054–0.400	0.093	769.6
URB 1	0.023	0.05	0.093	0.03	0.070	0.02	0.036–0.100	0.008	304.3
URB 2	0.050	0.10	0.440	0.50	0.390	0.30	−0.20–0.99	0.127	780.0
URB 3	0.023	0.05	0.130	0.10	0.110	0.10	−0.027–0.240	0.085	465.2
URB 4	0.000	0.00	0.160	0.20	0.160	0.20	−0.17–0.49	0.218	-
**Rural**	0.255	0.50	2.930	1.50	2.670	1.00	1.09–4.25	0.013	1049.0
RUR 1	0.068	0.10	0.000	0.00	−0.068	0.10	−0.28–0.15	0.391	−100.0
RUR 2	0.340	0.70	5.310	2.70	4.970	2.10	1.58–8.36	0.019	1461.8
RUR 3	0.000	0.00	0.520	0.40	0.520	0.40	−0.086–1.130	0.072	-
RUR 4	0.510	1.00	2.180	1.30	1.670	0.60	0.79–2.55	0.009	327.5
RUR 5	0.300	0.60	2.250	1.10	1.940	0.50	1.13–2.76	0.005	650.0

CI, confidence interval; DDD, defined daily dose; s.d., standard deviation; pop, population; Diff, difference; Prov, provincial.

Pre-mean = mean consumption per quarter pre-policy implementation; Post-mean = mean consumption per quarter post-policy implementation. Diff-Mean = mean difference between the post-policy consumption and the pre-policy consumption. Percentage (%) change = ([post-consumption – pre-consumption] ÷ pre-consumption) × 100.

As shown in Table 1, sodium valproate consumption increased by 3.3% (*p* = 0.255), 7.8% (*p* = 0.255) and 1.5% (*p* = 0.701) for the province, urban and rural areas, respectively; however, these increases were not statistically significant. Similarly, in the sub-analysis, none of the urban substructures (URB 1 to URB 4) or rural districts (RUR 1 to RUR 5) showed a significant change in valproate consumption.

As presented in Table 2, lamotrigine consumption increased significantly for the province (30.7%; *p* = 0.020) and urban (54.5%; *p* = 0.002) but not for rural (26.1%; *p* = 0.108) areas. Increase of lamotrigine consumption in URB 1, 2 and 3 was statistically significant (*p* < 0.001; *p* = 0.014; *p* = 0.048); however, RUR 3 was the only rural district that showed significance (*p* = 0.004).

The consumption of levetiracetam, as shown in Table 3, increased significantly in the province (*p* = 0.016) and the rural area (*p* = 0.013). It was greater in rural areas compared with urban areas. The very low level of consumption of levetiracetam resulted in statistically significant increases; however, overall, the consumption remained very low compared with valproate and lamotrigine consumption.

## Discussion

Analysis of aggregate data is an efficient and useful signal to assess whether more resource-intense investigations or interventions are warranted. The key findings of this study, demonstrating little change in valproate consumption in the study population, raise important issues with implementing the valproate policy within WCGH facilities.

The provincial analysis that included 99% of public health facilities (242/245), as well as the individual cluster or geographical analysis, did not indicate a significant change in valproate consumption during one year after the valproate policy implementation. Furthermore, as tertiary-, regional-, specialist hospitals, etc., were excluded from the geographical analysis, referral of patients to level-2 or -3 facilities was not significant to change the direction of the geographical results after policy implementation. For lamotrigine, there was a significant increase in consumption between pre- and post-policy periods for the province and the urban areas but not for the rural areas. However, the consumption for lamotrigine (32 DDDs) still remained far below the consumption for valproate (209 DDDs). A possible explanation for the more stable use of sodium valproate and the increase in lamotrigine could be that new patients may be initiated on lamotrigine rather than those being switched from sodium valproate to lamotrigine. Previously, lamotrigine initiation was restricted to specialist prescribers, but with the implementation of the policy, the initiation of lamotrigine was extended to medical officers, which generally results in increased use of a medicine. Together with the epilepsy policy, lamotrigine was introduced with a prospective safety monitoring tool and training to address prescribers’ hesitancy in initiating lamotrigine.^26^ Thus, there was more awareness around the use of lamotrigine during policy implementation. The slow uptake of lamotrigine in rural areas may be because of access to doctors and challenges with providing training in rural areas. Clinics are usually nurse driven, whereas CHC/CDCs are doctor driven and have pharmacies. The only rural district that showed a significant increase in the use of lamotrigine was RUR 3, which has a higher number of CHC/CDCs as well as a higher CHC/CDC to clinic ratio compared with other rural districts. Although the increase in levetiracetam was significant and the percentage change was high, this was based on very low levels of use; hence, consumption was still relatively limited. This was expected as the number of patients unable to take lamotrigine (e.g. due to non-adherence, intolerance or treatment failure) would be few. Side effects such as irritability, aggression and suicidal and psychotic episodes limit the preference of levetiracetam use in practice.^7,22^

The introduction of the valproate policy was multifaceted with detailed information on alternatives to valproate, appropriate doses, important counselling information, forms for the acknowledgment of risk for patients and clinicians and was introduced with training sessions and a presentation for those unable to attend the training sessions. In addition, an electronic warning label, generated on dispensing valproate, was implemented. The aim was to remind patients of the harms of valproate, and that they could approach the doctor for safer alternatives. Prescribers’ concerns with lamotrigine were addressed during training sessions, and a monitoring tool for pharmacists for the safe initiation of lamotrigine was implemented concurrently. The results of this study indicated that the valproate policy was more complex to implement with several barriers to overcome.

A local study attributed tardiness of switching WOCBA from valproate to safer alternatives to fear of breakthrough seizures, challenges in initiating lamotrigine, overburdened health system and inadequate access to specialists.^21^ Similarly, in this study, the bulk of patients on valproate were at PHC level/Level 1 under the management of medical doctors and having limited access to specialists. Lamotrigine requires slow titration over an approximately 8-week period to prevent serious side effects, which is a major challenge in a resource-constrained health system and for patients who may default therapy. Understandably, this would create prescriber hesitancy. Consequences of uncontrolled seizures have a significant impact on livelihoods, and therefore, changing to another antiepileptic, especially if controlled on valproate, requires not only the prescribers’ decision but also the patient’s consent and readiness. Stable patients usually have 6-monthly doctor consultations, and thus, this would also delay the transition of patients being changed from valproate to safer options.

The slow transition from sodium valproate to lamotrigine requires exploration. In Switzerland, the Dear healthcare professional letter from the European Medicines Agency led to a decrease in sodium valproate initiation in bipolar mood disorders but not in the treatment of epilepsy.^27^ Furthermore, in a study conducted in the United Kingdom, clinicians were surveyed to monitor the use of risk acknowledgement forms with sodium valproate therapy, one year after the valproate risk acknowledgement form was implemented by the Medicines Healthcare products Regulatory Agency.^28^ Lamotrigine followed by levetiracetam was the most frequently prescribed antiepileptic replacing valproate. The risk acknowledgement form for WOCBA without intellectual disability reflected that 43.5% clinicians reported that ‘most’ of their patients continued with valproate, while an additional 25.0% reported that ‘all’ of their patients continued with valproate. Levetiracetam (33%) and lamotrigine (43%) were more associated with deterioration in seizure control compared with patients continuing on sodium valproate (7%).^28^ Both studies show hesitancy in transitioning from valproate to alternative therapies, reflecting the complexity of our valproate policy recommendations, not only in our setting but also in high-income countries. Analysing the rate of completion of the acknowledgement of risk forms of the clinicians and patients in our population would shed some light on the slow transition from valproate to safer alternatives for WOCBA and patient readiness. Also, the form could be amended to include pertinent details of reasons for not switching to valproate alternatives.

The change from sodium valproate to alternative therapy is complex and might be associated with a reduction in seizure remission.^28,29^ The relationship between sodium valproate avoidance and switch to alternative therapy in WOCBA was investigated over the period 1980–2018 in Italy.^29^ The study, as with the United Kingdom study,^28^ found that patients who switched from valproate to alternative therapies experienced a clinical worsening, and switching back to valproate was more often associated with seizure remission. The study, therefore, suggested valproate avoidance or switch to alternative therapy might be associated with a reduction in seizure remission.^29^ It is important to understand our local context, and therefore, monitor the patient outcomes of our policy recommendations.

Although the valproate policy involved many facets, including monitoring aspects, a more robust monitoring tool may be required. Policy monitoring is essential to determine improvement in service delivery and health outcomes.^30^ Behavioural change is not only a key influential factor to the success of policy change but also a complex process with barriers and facilitators that need to be explored.^27^ Interventions that facilitate behavioural change include education, incentives, multifaceted interventions, audits and monitoring.^31,32^ A feasible monitoring tool that includes the assessment of the acknowledgement of risk forms, patients’ outcomes and valproate switching details would inform on barriers and policy success. In addition, medicine use evaluations (MUEs) should be strengthened as a promoter of behavioural change. An MUE follows a continuous quality improvement cycle to assess appropriate medicine use and includes an investigational and intervention phase.^33^ Pharmacists usually lead MUEs in facilities, provide feedback and conduct educational sessions on MUEs. Strong governance such as provided by a pharmaceutical and therapeutics committee is important to monitor findings and guide corrective actions.

## Strengths and limitations

In South Africa, we face various challenges that prohibit the easy assessment of appropriate patient care. Some of these examples include paper-based medical notes, lack of continuity of prescribers, constrained resources, nonintegrated digital medical records, etc. Therefore, as an initial investigation, this study followed a manageable, reliable method requiring limited resources. The study is easily reproducible for continued monitoring. The electronic aggregate data included in the study was from a reliable source and included 99% of the public health facilities. These primary health care facilities and hospitals provide services to more than three-quarters of the Western Cape population.

The data included medicine supplied to facilities, rather than what was dispensed to patients. Working with this type of aggregate data does not allow for differentiation between age, gender, pregnant women, WOCBA and diagnosis. As this was an aggregate dataset, we could not adjust for potential confounders or explore barriers or facilitators for prescribing behaviours. The data, however, provide a strong and urgent signal for more detailed investigations and interventions to address the safe use of valproate in WOCBA.

## Conclusion

In the Western Cape public sector, the implementation of a policy recommending alternatives for valproate in WOCBA did not significantly change the consumption of sodium valproate. Although the use of lamotrigine and levetiracetam increased significantly, consumption remained low compared with the sodium valproate consumption. The findings from this study warrant further investigation focusing on WOCBA to substantiate this signal and shed light on limits of the current data analysis.
